# Qualitative and quantitative assay of glucose 6 phosphate dehydrogenase in patients attending tertiary care center

**DOI:** 10.1186/s13104-020-05145-8

**Published:** 2020-06-22

**Authors:** Uday Sharma, Satyendra Mishra, Narayan Gautam, Badri Kumar Gupta

**Affiliations:** 1 Universal College of Medical Sciences, Bhairahawa, Nepal; 2 Department of Pathology, Universal College of Medical Sciences, Bhairahawa, Nepal; 3 Department of Biochemistry, Universal College of Medical Sciences, Bhairahawa, Nepal; 4 Department of Pediatrics, Universal College of Medical Sciences, Bhairahawa, Nepal

**Keywords:** Glucose 6 phosphate dehydrogenase, Hemolytic disorders, Methemoglobin reduction test, Spectrophotometric assay

## Abstract

**Objectives:**

The study was carried out with the aim to find out the frequency of Glucose 6 phosphate dehydrogenase (G6PD) deficiency among the patients attending the hospital and to rationalize the qualitative methemoglobin reduction test in reference to the quantitative spectrophotometric assay. Timely screening of the patients for G6PD with appropriate screening method can play an important role in preventing hemolytic crisis that arises from therapeutic use of oxidative drugs like primaquine.

**Result:**

The frequency of G6PD deficient cases was 3% by both of the employed tests. The mean ± SD of G6PD activity in the patients under study was 15.34 ± 4.7 IU/g Hb in males and 16.01 ± 3.74 IU/g Hb in females. G6PD activity was positively associated with reticulocyte count (r = 0.289, *p* value = 0.004) and negatively with mean corpuscular hemoglobin concentration (r = −0.220, p-value = 0.028). The correlation of red blood corpuscular count and G6PD was statistically significant (p-value = 0.048).

## Introduction

Glucose-6-phosphate dehydrogenase (G6PD) is a rate limiting enzyme of the pentose phosphate pathway and is closely associated with the hemolytic disorders among patients receiving anti-malarial drugs, such as primaquine [1]. G6PD deficiency is an x-linked recessive hereditary disorder [2]. This disorder affects 200–400 million people worldwide. The G6PD gene codes for an enzyme that catalyzes the reaction that converts nicotinamide adenine dinucleotide phosphate (NADP^+^) into NADPH which is a reduced form, in pentose phosphate pathway [3].

G6PD deficiency has been classified into five classes according to the severity of the deficiency of the enzyme:

Class I deficiency is defined as severe deficiency and is associated with chronic non-spherocytic hemolytic anemia. Class II deficiency is also defined as severe deficiency with enzyme activity of 1%–10% of normal activity. Class III deficiency are moderately deficient and their enzyme activity is 10%–60% of normal activity. Class IV and Class V individuals have normal and increased activity with an enzyme activity of 60%–150% and over 150%, respectively [4].

At the public health level, at least simple screening methods for G6PD deficiency are needed in order to detect a hemizygous males and homozygous females to avoid an acute hemolytic crisis, newborn hemizygous males and homozygous females to detect neonatal jaundice and early treatment and lastly, to detect heterozygous females, to give specific advice about the care of their newborn male infants [5].

Not only primaquine but a large spectrum of drugs can induce hemolysis in G6PD deficient individuals [6]. Ingestion of fava beans (*Vicia faba*) has been associated with hemolytic anemia in G6PD deficient individuals since a very ancient period of time [7]. Exposure of infant or mother to oxidant drugs or without exposure to such drugs leading neonatal jaundice due to G6PD deficiency can even lead to kernicterus [8].

In chronic Non-Spherocytic Hemolytic Anemia, the affected individuals have a moderately severe hemolytic anemia in association with reduced activity of erythrocyte G6PD [9].

For the identification of G6PD deficiency in patients, there are five types of phenotypic tests:

I Direct enzyme activity assay: Spectrophotometry and Beutler’s fluorescent spot test.
II Indirect assay: Methemoglobin reduction test and brilliant cresyl blue or formazan ring tests.
III Cytofluorometric assay Hirono-1-methoxy PMS Sephadex method and WST8/1 methoxy PMS method.
IV Rapid tests Hirono-1-methoxy PMS Sephadex method and WST8/1 methoxy PMS method.
V Rapid point-of-care tests: Binax Now G6PD and Carestart G6PD [10, 11].

Screening of the population is desirable in the regions with a G6PD deficiency prevalence ≥ 3–5% before the use of contraindicated drugs (such as primaquine) in order to avoid hemolytic complications [12].

## Main text

### Method

This hospital based cross sectional and comparative study was conducted in total of 100 patients attending the hospital with clinical as well as laboratory suspicion like fever and shortness of breath, hyperbilirubinemia, jaundice after antimalarial drug therapy, peripheral smear showing bite cells, Heinz bodies and other hemolytic blood picture were included. Patients of age more than 60 years of age were excluded from the study.

Study population and sample size was determined by using the formula: $n = \frac{{Z^{2} PQ}}{{D^{2} }}$.

n = (1.96)^2^ × 0.07 × 0.93/(0.0025 = 100 where, n = sample size, z = critical value = 1.96, P = prevalence of disease = 7%, Q = without disease (1-P), D = allowance error (5%).

The hematological parameters including hemoglobin concentration, Red blood corpuscular (RBC) count, Packed Cell Volume (PCV), Mean Corpuscular Volume (MCV), Mean Corpuscular Hemoglobin (MCH) and Mean Corpuscular Hemoglobin Concentration (MCHC) of the patient were obtained from Hematology analyzer 5 parts (Beckman coulter, DxH 520).

### Spectrophotometric assay of G6PD

G6PD in RBCs is released by lysing agent present in the reagent. The G6PD released from the red cells catalyzes the Glucose-6-phosphate with reduction of NADP to NADPH. The rate of reduction of NADP to NADPH is measured as an increase in absorbance at 340 nm produced in the reaction catalyzed by the enzyme which is proportional to the G6PD activity in the sample [13]. Coral G6PD assay kit (Clinical System, Bambolim complex, Goa, India) was used and the procedure provided in the manual with the kit was followed. The absorbance was taken 2 min after reaction mixture was added with blood and final value was calculated by multiplying absorbance (∆A) with factor 4778 in Human Semi-automated analyzer divided by hemoglobin concentration of patients. The normal value of the G6PD activity at 37 °C is 6.4–18.7 IU/g Hb. At low, medium and high G6PD, intra assay coefficient of variation (CV) were 5.91%, 4.98% and 4.83% and inter-assay CV were 7.85%, 8.43% and 6.35%.

### Methemoglobin reduction test

The action of nitrite on red cells results in formation of an oxidized form, methemoglobin, and in the presence of methylene blue, methemoglobin is reduced to hemoglobin through the oxidative pathway. The absence of glucose 6 phosphate dehydrogenase is determined by unchanged brown colored methemoglobin after addition of methylene blue and incubation [8]. 2.0 ml of blood was taken in the test tubes marked with ‘positive control’, ‘negative control’ and ‘test’. 0.1 ml of sodium-nitrite-glucose solution and 0.1 ml of methylene blue solution was added in test tubes marked ‘positive control’ and ‘test’ and mixed. No reagent was added in the negative control tube. After 3 h of incubation, 0.1 ml of solutions from each tube was transferred to clean and labelled new test tubes. The volume was made up to 10 ml and observed for the comparison of ‘test’ with ‘positive control’ and ‘negative control’. If the ‘test’ was clear red similar to negative control, it was interpreted as negative and if it was brown, similar to positive control, it was interpreted as positive for G6PD deficiency. The controls were prepared from the same blood samples that were being tested or the samples from healthy individuals with hemoglobin concentrations not differing more than ± 0.5 g/dl than that of the samples being tested.

Statistical analysis was done using SPSS IBM ver. 22, New York. The parametric data were expressed in mean ± SD. Categorical data were expressed as frequencies with corresponding percentages and evaluated using Chi Square test with level of significance set to 0.05 for all tests and P-value < 0.05 was considered significant.

### Results

Table 1 shows the results from both the implied tests for G6PD. Three deficient cases had 2.46 IU/g Hb, 3.98 IU/g Hb and 2.21 IU/g Hb G6PD activity respectively by spectrophotometric assay.

**Table 1:** Comparison of two methods (Methemoglobin Reduction and Spectrophotometry) for Assay of G6PD

Results	Methemoglobin reduction test	Spectrophotometric assay	Mean ± SD
Deficient	3 (3%)	3 (3%)	2.88 ± 0.95
Normal	97 (97%)	90 (90%)	15.38 ± 2.60
Hyperactive	Not applicable	7 (7%)	25.07 ± 2.95
Total	100 (100%)	100 (100%)	

Table 2 shows the mean value and standard deviation of different variables under study with differentiation between two sexes.

**Table 2:** Mean value and standard deviation of study variables

Variables	Sex	Mean ± SD	P-value
0–14 years	M	5.92 ± 3.40	0.06
	F	7.9 ± 4.38	
> 14 years	M	37.06 ± 16.60	
	F	33.95 ± 14.25	
	Average	28.78 ± 18.25	
Hb (g/dl)	M	9.06 ± 2.37	0.95
	F	8.13 ± 2.37	
	Average	8.59 ± 2.37	
RBC (millions/mm^3^)	M	3.28 ± 0.97	0.19
	F	3.00 ± 1.13	
	Average	3.14 ± 1.05	
PCV (%)	M	27.51 ± 7.64	0.71
	F	23.34 ± 7.69	
	Average	25.42 ± 7.66	
MCV (fl)	M	85.01 ± 15.62	0.36
	F	85.32 ± 16.83	
	Average	85.16 ± 16.22	
MCH (pg)	M	28.15 ± 5.89	0.41
	F	27.43 ± 6.08	
	Average	27.79 ± 5.98	
MCHC (g/dl)	M	32.40 ± 3.31	0.09
	F	31.97 ± 1.45	
	Average	32.18 ± 2.38	
Reticulocyte count (%)	M	1.56 ± 0.87	0.62
	F	1.69 ± 0.93	
	Average	1.62 ± 0.90	
G6PD activity (IU/g Hb)	M	16.01 ± 3.74	0.68
	F	15.67 ± 4.22	
	Average	15.34 ± 4.7	

Table 3 shows the Pearson’s correlation between different variables with their level of significance.

**Table 3:** Correlation among study variables

Variables	Hb	PCV	MCV	MCH	MCHC	Reticulocyte count
Age						
r	0.052	0.064	− 0.212	− 0.242	− 0.022	− 0.241
p	0.609	0.524	0.034*	0.015*	0.831	0.016*
RBC						
r	0.786	0.821	− 0.524	− 0.515	− 0.270	− 0.081
p	0.001**	0.001**	0.001**	0.001**	0.007**	0.422
G6PD						
r	− 0.100	− 0.098	− 0.042	− 0.118	− 0.220	0.289
p	0.321	0.332	0.676	0.241	0.028*	0.004**

*r* Pearson correlation, *p* Significance (two-tailed), *(correlation is significant at 0.05 level, two tailed), **(correlation is significant at 0.01 level, two tailed)

### Discussion

This study attempted to check the reliability of qualitative method of testing G6PD by methemoglobin reduction test with reference to the spectrophotometric method and also to find out the frequency of deficient cases among the patients attending UCMS-TH, Bhairahawa, Nepal.

Hundred subjects were studied in our research and the frequency of G6PD deficient subjects was found to be 3% which was in the same patients by both the assays. In a study done by Oni G et al. 60% were male subjects and 40% were female subjects with frequency of G6PD deficient males 40 and that of females 35 in contrast to our study in which, among 52% females and 48% males and all 3 of the deficient individuals were males [14].

Thilakarajan S et al. stated, in their study, that the methemoglobin reduction test was able to pick heterozygous females. Their study tested subjects only with methemoglobin reduction test and suggested to analyzing its efficacy with a simultaneous enzymatic assay. In contrast to their study, the present study has adopted both the methods for testing G6PD assay. But in our study, none of the female subjects were found to be G6PD deficient hence we couldn’t predict if Methemoglobin Reduction test could analyze heterozygous females or not [15].

The frequency of positive subjects in our study was similar to that found in Dhanusa district in a previous study by Lamichanne N et al. where it was 3.1%. While it was different from prevalence in other districts like Jhapa where it was 9.8% and in Morang district where it was 5.8%. Our study was hospital based unlike their study. The samples in our study were suspected cases of G6PD deficient patients attending UCMS-TH only which could be low as compared to the susceptible patients of G6PD deficiency regions [16].

The mean ± SD of G6PD activity in the sample population was 15.69 ± 4.23 IU/g Hb in our study which is quite different than that of Kim S et al. who projected 10.9 ± 4.6 IU/g Hb. Since the study done by them was in a different region of Asia i.e. in Cambodia and G6PD enzyme activity may vary geographically, this difference might have been observed. Moreover, their method of assay was different from our study. They performed the tests using CareStart™, a rapid diagnostic test method for G6PD screening while ours was according to G-Six Kinetic Assay, Tulip Group, India, by Spectrophotometric assay [17].

The male subjects having G6PD deficiency outnumbered the number female subjects in our study, similar to the study by Das K P et al.in which all the deficient subjects were males and the study conducted by Gautam N et al. in which 81.8% of the deficient subjects were males with overall 11% deficiency of G6PD in southwestern region province number 5 of Nepal [18, 19]. This suggests the fact that G6PD deficiency is an x-linked genetic disorder and males are more prone to be affected than females.

In our study, the frequency of subjects having increased enzyme activity was 7% which is very similar to the result obtained by Khim N et al. in which they have 5.1% of subjects considered in Class V which WHO classifies as a class of individuals having an increased activity of G6PD enzyme [20, 21]. According to Domingo G J et al. high count of young red cells or high leukocyte count might result in high G6PD activity because G6PD level is higher in these cells which supports our study in a way that all the subjects with hyperactivity of enzyme were having an increased level of reticulocyte count [22].

The reticulocyte count was higher in the G6PD deficient subjects similar to the result of Al-Nood AH et al. but in our study, the reticulocyte count was higher in all the subjects with hyperactive enzyme status. The mean ± SD of reticulocytes was 1.62 ± 0.90 and P-value was 0.004 [23]. In our study 25% of the subjects were having high reticulocyte count and remaining 75% had a normal count.

The G6PD activity was statistically correlated with RBC count with P-value of 0.048 in our study while with PCV there was no significant correlation found. Since the G6PD deficiency precipitates the hemolysis with other factors, it might have caused the RBC count to be significantly reduced. The statistical association between hemoglobin level and G6PD was not observed in the present study.

### Conclusion

Not very frequent but there is a presence of G6PD deficiency in patients attending UCMS-TH. The methemoglobin reduction test is convenient and cheaper. Although it is a time consuming method, for the mass screening it could be very easy because several samples can be tested at once. The positive results given by this method were very satisfying with Spectrophotometric assay in our study even though it was not able to generate exact enzyme activity in the sample. It is advisable to screen suspected patients with methemoglobin reduction test before initiating oxidative drug therapy.

## Limitations

The study would have been more effective if we had studied partially deficient females that could generate the actual efficacy of methemoglobin reduction test. And the current study is hospital based while, community based study with larger sample size is better for assessing the exact burden of G6PD deficiency.

## Data Availability

The data set that was used and analyzed in the current study are available from the corresponding author upon reasonable request.

## Abbreviations

G6PD: Glucose 6 phosphate dehydrogenase
Hb: Hemoglobin
UCMS-TH: Universal College of Medical Sciences-Teaching Hospital
RBC: Red blood corpuscular
PCV: Packed cell volume
MCV: Mean corpuscular volume
MCH: Mean corpuscular hemoglobin
MCHC: Mean corpuscular hemoglobin concentration
NADP: Nicotinamide adenine dinucleotide phosphate
